# Supplementary material for: FIGO 2018 Staging for Cervical Cancer: Influence on Stage Distribution and Outcomes in the 3D-Image-Guided Brachytherapy Era
Source: Cancers (Basel). 2020 Jul 2;12(7):1770. doi: 10.3390/cancers12071770 (PMC7408064; doi:10.3390/cancers12071770)
Supplement: Supplementary file 1 [file cancers-12-01770-s001.docx]

Supplementary Files

FIGO 2018 Staging for Cervical Cancer: Influence on Stage Distribution and Outcomes in the 3D-Image-Guided Brachytherapy Era

Kento Tomizawa, Takuya Kaminuma, Kazutoshi Murata, Shin-ei Noda, Daisuke Irie, Takuya Kumazawa, Takahiro Oike and Tatsuya Ohno


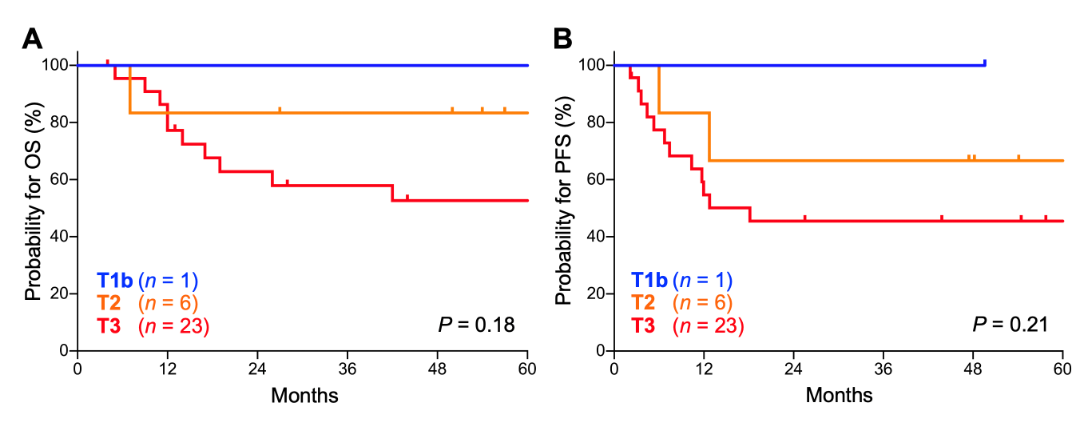


**Figure S1.** Kaplan–Meier estimates of overall survival (OS) (**A**) and progression-free survival (PFS) (**B**) for stage IIIC2_r_ patients with squamous cell carcinoma of the cervix according to the International Federation of Gynecology and Obstetrics (FIGO) 2018 staging criteria stratified by T stage based on the classification of the Union for International Cancer Control, 7th edition. *p* values calculated from the log-rank test are shown.

| 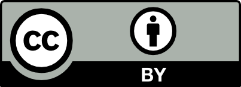 | © 2020 by the authors. Licensee MDPI, Basel, Switzerland. This article is an open access article distributed under the terms and conditions of the Creative Commons Attribution (CC BY) license (http://creativecommons.org/licenses/by/4.0/). |
| --- | --- |
